# Emergence of functionally aberrant and subsequent reduction of neuromuscular connectivity and improved motor performance after cervical spinal cord injury in Rhesus

**DOI:** 10.3389/fresc.2023.1205456

**Published:** 2023-06-12

**Authors:** Gregory Wai, Sharon Zdunowski, Hui Zhong, Jessica L. Nielson, Adam R. Ferguson, Sarah C. Strand, Rod Moseanko, Stephanie Hawbecker, Yvette S. Nout-Lomas, Ephron S. Rosenzweig, Michael S. Beattie, Jacqueline C. Bresnahan, Mark H. Tuszynski, Roland R. Roy, V. Reggie Edgerton

**Affiliations:** ^1^ Departments of Integrative Biology and Physiology, University of California, Los Angeles, Los Angeles, CA, United States; ^2^ Rancho Los Amigos National Rehabilitation Center, Rancho Research Institute, Downey, CA, United States; ^3^ Department of Psychiatry & Behavioral Sciences and the Institute for Health Informatics, University of Minnesota, Minneapolis, MN, United States; ^4^ Brain and Spinal Injury Center, Department of Neurological Surgery, University of California, San Francisco, San Francisco, CA, United States; ^5^ California National Primate Research Center, University of California, Davis, Davis, CA, United States; ^6^ Department of Clinical Sciences, College of Veterinary Medicine and Biomedical Sciences, Colorado State University, Fort Collins, CO, United States; ^7^ Veterans Administration Medical Center, La Jolla, CA, United States; ^8^ Department of Neuroscience, University of California, San Diego, La Jolla, CA, United States; ^9^ Brain Research Institute, University of California, Los Angeles, Los Angeles, CA, United States; ^10^ Institut Guttmann, Hospital de Neurorehabilitacio, Universitat Autonoma de Barcelona, Badalona, Spain; ^11^ Neurorestoration Center, University of Southern California, Los Angeles, CA, United States

**Keywords:** muscle activity patterns, motor performance, spinal plasticity, spinal cord injury, EMG

## Abstract

**Introduction:**

The paralysis that occurs after a spinal cord injury, particularly during the early stages of post-lesion recovery (∼6 weeks), appears to be attributable to the inability to activate motor pools well beyond their motor threshold. In the later stages of recovery, however, the inability to perform a motor task effectively can be attributed to abnormal activation patterns among motor pools, resulting in poor coordination.

**Method:**

We have tested this hypothesis on four adult male Rhesus monkeys (*Macaca mulatta*), ages 6-10 years, by recording the EMG activity levels and patterns of multiple proximal and distal muscles controlling the upper limb of the Rhesus when performing three tasks requiring different levels of skill before and up to 24 weeks after a lateral hemisection at C7. During the recovery period the animals were provided routine daily care, including access to a large exercise cage (5' × 7' × 10') and tested every 3-4 weeks for each of the three motor tasks.

**Results:**

At approximately 6-8 weeks the animals were able to begin to step on a treadmill, perform a spring-loaded task with the upper limb, and reaching, grasping, and eating a grape placed on a vertical stick. The predominant changes that occurred, beginning at ∼6-8 weeks of the recovery of these tasks was an elevated level of activation of most motor pools well beyond the pre-lesion level.

**Discussion:**

As the chronic phase progressed there was a slight reduction in the EMG burst amplitudes of some muscles and less incidence of co-contraction of agonists and antagonists, probably contributing to an improved ability to selectively activate motor pools in a more effective temporal pattern. Relative to pre-lesion, however, the EMG patterns even at the initial stages of recovery of successfully performing the different motor tasks, the level of activity of most muscle remained higher. Perhaps the most important concept that emerges from these data is the large combinations of adaptive strategies in the relative level of recruitment and the timing of the peak levels of activation of different motor pools can progressively provide different stages to regain a motor skill.

## Introduction

Significant levels of recovery of upper limb function can occur after a cervical spinal cord injury and the severity of the deficits are largely a function of the level of the cervical injury and the severity of the lesion. We have reported that after a unilateral cervical lesion (C7) in nonhuman primates (1–3), there is extensive branching of the corticospinal axons projecting across the midline to the injured side. We also showed that recovery of locomotor function was substantial and there was a gradual change in the coordination patterns of agonist and antagonist muscle activity. The present paper examines further details of the post-lesion recovery of motor function. We reasoned that to regain some systemic-level understanding of this reparative process every movement is defined essentially by two basic physiological variables, i.e., the net level of excitation of each motor pool and the precision of the coordination among the motor pools that generate the motor task. Given the fundamental importance of these two variables, we systematically and comprehensively assessed the physiological properties that reflect these variables before and up to 24 weeks after a cervical lateral hemisection (C7) in the Rhesus.

One might predict that the loss of function after a cervical lesion would be due largely to a loss of the ability to activate the motor pools relevant for a particular motor task. This indeed appears to be the case during the very early post-lesion period, but during the subsequent stages of recovery several observations suggest that the reverse may be the case (4–11), i.e., an excessively high number of motor pools and motor units are recruited when performing a given task post-injury. This time course of events suggests the formation of functionally aberrant connections during the initial stages of recovery of movement followed by a gradual decrease in the functional connectivity with post-injury time.

Theoretically, the consequences of forming additional functionally aberrant connections at any point along the sensorimotor axis would be an excessive excitation of motor pools and an increase in instances of co-contraction. For example, electromagnetic stimulation of the motor cortex in human subjects after a spinal cord injury activates multiple muscles within a few days: a result rarely observed with the same stimulation parameters in uninjured individuals (4). In addition, unusually diverse combinations of muscles are activated after an incomplete spinal cord injury when the subjects attempt to voluntarily initiate a movement (9). A spatial reorganization of sensory-evoked potentials projecting to the sensorimotor cortex also can occur within hours after a peripheral nerve injury (12) or amputation of the distal tip of a digit (13). Similar observations have been observed in response to the stimulation of spinal networks via spinal epidural (14) or noninvasive transcranial stimulation (15) following a spinal cord injury in human subjects.

In the present study we quantified EMG patterns of proximal and distal flexor and extensor muscles in the upper limb of a nonhuman primate while performing a relatively automatic task, i.e., stepping on a treadmill, as well as during tasks requiring finer “conscious” control to include pulling a spring-loaded handle and removing a grape on a stick before and after a cervical lateral hemisection. Comparison of the performance of these different motor tasks was to determine whether any re-organization associated with aberrant connections would be more critical in performing a fine motor task vs. locomotion. These comparisons could have clinical implications as to whether any expanding functional connectivity could be more prominent within and/or among the supraspinal and spinal networks.

## Materials and methods

### Subjects

Four adult male Rhesus monkeys (*Macaca mulatta)* (6–10 years old, mean of 8.6 years; mean weight: 12.2 kg) were studied. All surgical and experimental procedures were carried out using the guidelines set by the Laboratory Animal Care (US National Institutes of Health Publication 85–23, revised 1985) and were approved by the Institutional Animal Care and Use Committee (IACUC) of the California National Primate Research Center (CNPRC) at the University of California, Davis. Specific protocols with regard to sleep, shelter, and nourishment are outlined in Nout et al. (1, 2).

### Task training and testing

Pre-lesion, the monkeys were trained to step on a treadmill twice/week. They also were trained to perform a handle pull and a grape on a stick (GOAS) task with their ipsilateral (lesion side) forelimb while in a restraint chair three times/week for 30-min/session. Each chair trial began with the limb in a trained resting position using a clear plexiglas partition to only allow access to the food reward with the arm on the impaired side and to serve as a signal for the subject to begin the task. The trial ended when the handle was pulled maximally and released, the grape was retrieved, or there was no retrieval response after 15 s. Food rewards were used for positive reinforcement. After the spinal cord hemisection (see below), the monkeys were retrained as soon as they were able to participate in the tasks 2–3 times/week for 30-min/session.

#### Treadmill task

The monkeys were trained to walk quadrupedally on a motor-driven treadmill. A plexiglass enclosure was utilized to keep the animal in position while allowing video recording of their movements. Each training session consisted of 8 trials (two trials at 0.45, 0.89, 1.34, and 1.79 m/sec, 30 s/trial) with approximately one min of rest between each trial. Post-operatively, the maximum treadmill speed was adjusted according to each monkey's level of recovery, not to exceed 1.79 m/sec. The monkeys were stepped until they performed at least 10 consecutive and consistent steps at each speed with the contralateral hindlimb or stepped a maximum of 45 s.

#### Grape on a stick task

The monkeys were required to retrieve a food item (usually a grape) attached to the top of a vertical post that was positioned at about maximum reach length and shoulder height. The post also was anchored at the center of a platform containing a force sensor with six degrees of freedom. Ten trials were performed during each session. For five of these trials, a funnel (base diameter, 7.6 cm; height, 8.3 cm) was placed over the bottom of the post to promote retrieval of the grape using a pincer-grip and to prevent substitute forms of grasping. The remaining five trials were conducted without the funnel. A successful trial was defined as getting the food item to the mouth within 15 s after the food item was presented. No attempt to reach, failure to remove the item within 15 s, or removal and drop before reaching the mouth were defined as unsuccessful trials and subsequently not analyzed.

#### Handle pull task

The monkeys were required to pull a round handlebar (11.4 cm long × 6.4 cm wide × 0.64 cm diameter) attached to a plastic guide equipped with springs of differing tensions (20 N—easy; 60 N—medium; 98 N—difficult; and 107 N—very difficult). The handle was positioned horizontally or vertically (5 trials in each position per spring). Progressively greater spring tensions were utilized post-lesion as the monkey recovered from the surgery. A transducer attached in series with the handle and spring was used to record force and this signal was synchronized with EMG recordings from multiple muscles (see below).

#### Exercise cage activity

All animals were placed 4–5 times per week in a large open wire-mesh cage (7 feet [*H*] × 10 ft [*W*] × 6 ft [*D*]) with a series of 4 ascending elevated perches on the back and side wall of the enclosure, and a series of food cups hanging diagonally at different heights (1.5, 2.7, 3.5, 4.3, 4.7 ft) along the front of the cage. Each session began with cage entry through a chute onto the lowest perch. A large sized Kong® (a hollow rubber toy filled with small food rewards) was placed on the top perch to encourage movement. Use of the impaired forelimb was encouraged by the need to use both hands to retrieve items from the Kong. Next the food cups on the front of the cage were loaded to encourage climbing and impaired limb use. Next, presentation of a large food item (e.g., an apple or orange), a task requiring both hands for support and manipulation during eating, was used to promote use of the impaired hand. Each session lasted approximately 30 min and the cage was located in the housing room.

### Surgical and post-operative procedures

All surgical procedures were performed under aseptic conditions and with the monkeys sedated intramuscularly with ketamine (1 mg/kg) and then anesthetized to a surgical state with isoflurane gas to effect. Body temperature, heart rate, blood gases, respiration rate, and indirect blood pressure were monitored and maintained within acceptable levels.

#### EMG electrode implantation

Approximately 6–8 weeks prior to the spinal cord lesion, an EMG telemetry system (Konigsberg Instruments, Pasadena, CA, USA) having 6 EMG leads was implanted in each monkey. A transmitter and battery pack were implanted and secured between the internal and external oblique muscles on the non-injured side. Two wires (one lead) were tunneled subcutaneously into each of the following muscles: triceps brachii (TB), biceps brachii (BB), pronator teres (PT), flexor digitorum superficialis (FDS), extensor digitorum communis (EDC), and flexor pollicis brevis (FPB) and implanted intramuscularly as described previously (1, 2).

#### Spinal cord hemisection

A partial laminectomy was performed between vertebral levels C5 caudal and C6. An incision was made in the dura to expose the spinal cord and a lateral hemisection was performed at spinal cord level C7 using a micro-blade mounted on a stereotaxic carrier as described previously (1, 2).

#### Post-operative care

An analgesic (oxymorphone: 0.15 mg/kg) was given thrice daily for 3 days and an antibiotic (cephazolin: 25 mg/kg) twice daily for 7 days. The monkeys were returned to their home cage after recovery from surgery: the home cages were lined with fleece pads and porous rubber mats. Monkeys were monitored every 2–4 h and were given preferred food items to encourage them to sit up, reach, and stand.

### EMG and video recordings

Video and EMG were recorded during all task pre-lesion and approximately 6, 8, 10, 12, 16, 20, and 24 weeks post-lesion. Video was captured at 100 frames per second and edited using Simi Motion software (Simi Realty Motion Systems, Unterschleissheim, Germany). EMG was digitized at 2,000 Hz (National Instruments A/D board, Austin, TX) using custom software.

### Data processing

Raw EMG signals were band-pass filtered (20–500 Hz) and rectified. Cycle durations for the handle pull task were determined as the time between the activation of the BB in the ipsilateral forelimb and the return of the spring to its original position after the pull. For the GOAS task, the cycle duration was determined as the time between the onset of the reach of the ipsilateral forelimb (as seen on video) and the positioning of the grape in the mouth. If the monkey was unsuccessful in retrieving the grape, the end of the cycle was marked when the grape was dropped. Trials where the grape did not leave the stick were not marked. For the treadmill task, an average of ∼10 consecutive and most consistent steps from each trial were chosen for analysis. EMG burst durations, amplitudes, and integrals were determined as described previously (3).

The peak filtered and rectified EMG amplitude for each 0.5 msec time bin was plotted for the duration of each trial for the GOAS and handle pull tasks and for two consecutive step cycles for the treadmill locomotion. Joint probability distributions were generated to compare the EMG activation levels between selected muscle pairs for each trial for each task (16). We determined the percentage of data points exceeding 15% of a 2 mv maximum on both the x and y axes, i.e., 0.3 mv. Given that the majority of the data points are located less than 0.3 mv for both the x and y axes (baseline), these data points were excluded in the calculation of the percentage of co-activation (see Supplementary Figure S1).

### Statistics

To extract multivariate patterns across correlated muscle groups during recovery, we applied non-linear principal component analysis (NL-PCA) using optimal scaling method (17). We applied well-established rules for PCA extraction and PC retention (18). In brief, PCs were retained using 4 criteria: (1) the Kaiser rule, retaining PCs with eigenvalues >1.0 (19), (2) Scree plot (20), and (3) the over-determination of the factors (21), retaining factors with at least 3 loadings above. (4) PCs meeting all three criteria were examined and named using loadings above, thereby accounting for at least 20% of the variance. To test the hypothesis that multivariate PC patterns change together over time, we first performed a PCA on muscle recruitment and success metrics across all tasks and all timepoints then applied a general linear model (GLM), one-way analysis of variance (ANOVA) with Tukey's post-hoc test for significance to assess the impact of timepoint. Two-way GLM ANOVA was performed on PC scores to test the hypothesis that each task significantly changed over time for PC1. All analyses were performed in SPSS v.20 (IBM).

## Results

The alterations in the patterns of activation of proximal and distal extensor and flexor upper limb muscles were characterized over a period of up to 24 weeks post-injury. With the chronically implanted electrodes it was possible to make consistent comparisons of these patterns of activation throughout the stages of recovery after the lesion and to compare these patterns to those prior to the lesion. The activation patterns were compared when the animal was performing a relatively automatic type of movement, i.e., treadmill locomotion, and when performing tasks that required finer motor control, i.e., pulling a spring-loaded handle and removing a grape from the end of the stick that was instrumented with the 3-D force transducer. Several features were quantified that reflected the level of activation of each motor pool and the coordination among the motor pools during each task.

### EMG activity patterns during locomotion

**Figure 1:**
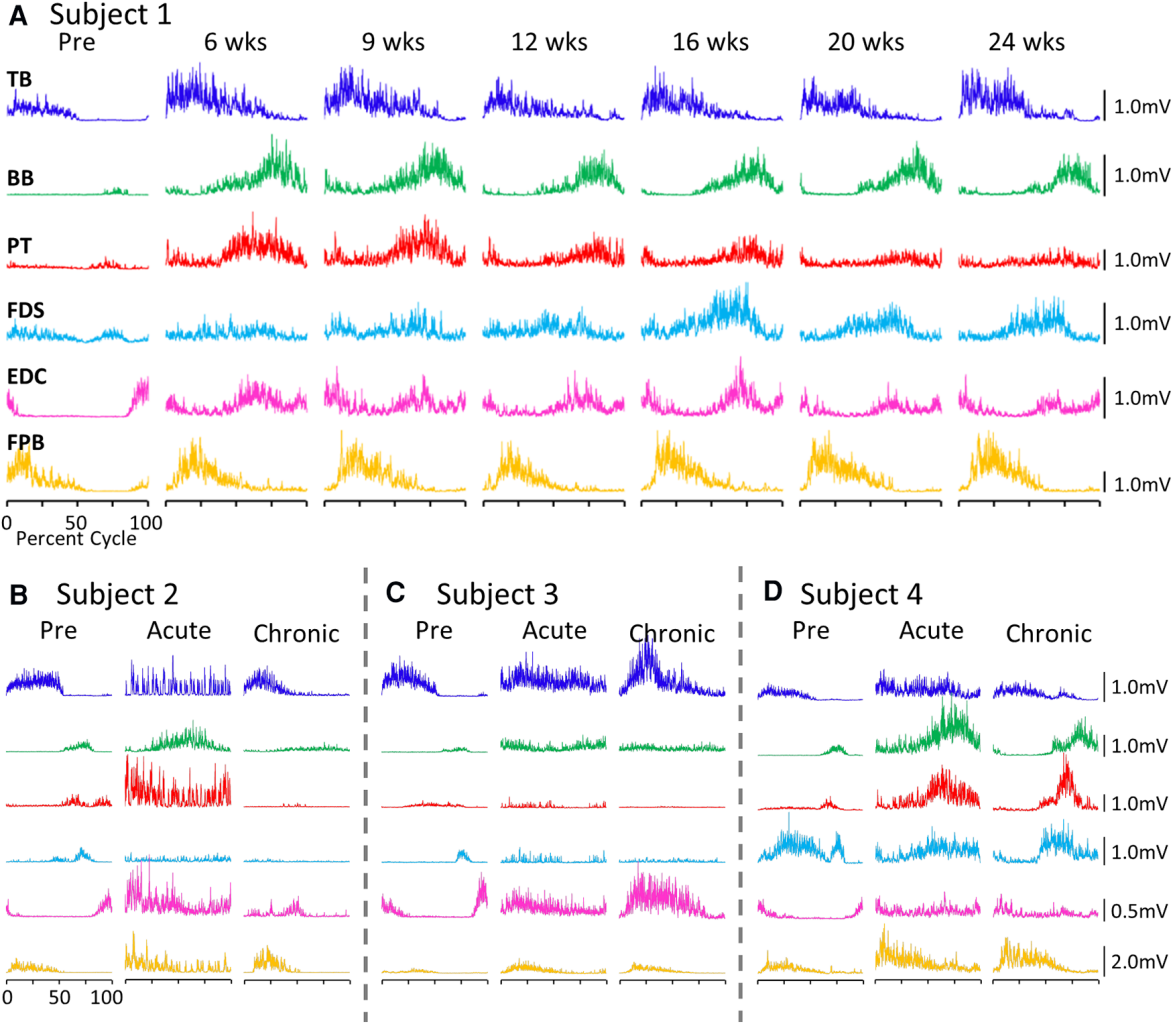
EMG patterns during locomotion. (**A**) Activation patterns (rectified EMG for ∼10 consistent, consecutive steps) of six upper limb muscles during locomotion on a treadmill at 0.89 m/s pre-lesion and 6 to 24 weeks post-spinal cord hemisection for Subject 1. Similar data for Subjects 2−4 pre-lesion and at an acute stage (6−8 weeks post-lesion) and a chronic (final recording session at 24 weeks post-lesion) stage are shown in (**B–D**). The beginning of the EMG trace is synchronized with the initiation of stance and all step cycle lengths are normalized. TB, triceps brachii; BB, biceps brachii; PT, pronator teres; FDS, flexor digitorum superficialis; EDC, extensor digitorum communis; FPB, flexor pollicis brevis.

The average EMG patterns for each muscle pre-lesion and up to 24 weeks post-lesion for Subject 1 are shown in Figure 1A. For the three remaining subjects similar data are shown for pre-lesion, at an acute stage (6–8 weeks post-lesion), i.e., the first time that the animal could make a minimal attempt at performing the motor tasks, and at the most chronic stage (24 weeks post-lesion), i.e., and the final recording session at approximately 24 weeks post-lesion (Figures 1B–D). Subjects 1, 2, and 3 had recovered sufficiently to resume locomotor testing at 6 weeks post-lesion, whereas Subject 4 was able to resume locomotor activity only after 9 weeks. At the end of the experimental period, the strategies used by the subjects to accomplish the task differed significantly (Supplementary Video S1). Subject 1 displayed only a slight limp and its gait most closely resembled that recorded pre-lesion. Subject 2 contacted the treadmill with the dorsal rather than the palmar surface of the hand with the ipsilateral forelimb during locomotion. Its body weight also seemed to be more heavily distributed toward the hindlimbs. Similar to Subject 2, Subject 3 contacted the treadmill with the dorsal surface of the ipsilateral hand while stepping on the treadmill. Subject 3 did not bear any weight with its ipsilateral hindlimb, i.e., the limb remained elevated during locomotion and resorted to a slight hopping motion on its contralateral side. Subject 4 also contacted the treadmill with the dorsal surface of its ipsilateral hand during stepping, but displayed a more even interlimb weight-bearing distribution and more normal stepping pattern.

**Figure 2:**
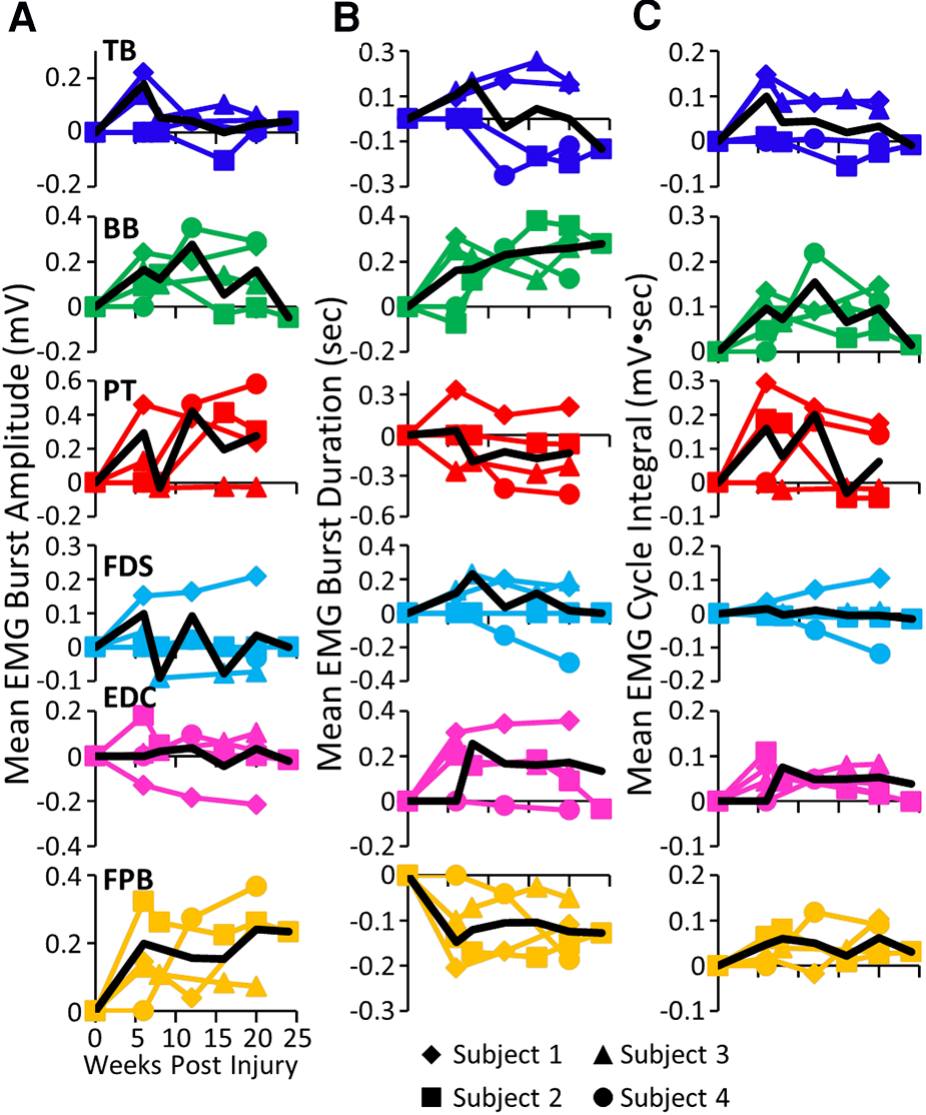
EMG characteristics during locomotion. Mean EMG burst amplitudes (**A**), durations (**B**), and integrals (**C**) and the overall mean (black traces) for each muscle and each subject for each time point. All post-lesion values are normalized to pre-lesion values. Muscle abbreviations, same as in Figure 1.

While all subjects were able to step on the treadmill and adjust to a range of speeds, there were both similarities and differences in the changes in EMG burst amplitudes during the recovery period across the subjects. The data in Figures 1, 2 demonstrate a rather consistent and marked increase in the level of activation of all motor pools early post-lesion in Subject 1 and generally in each of the other three subjects. In some cases, the EMG activity increased in the acute phase and eventually declined, even to a level less than was present before the lesion in some muscles. The other change that occurred in the activation patterns was the absence of a clear bursting pattern during a specific phase of a step. This was most evident in the acute phase while many of the motor pools recovered the ability to generate a more normal bursting pattern at the chronic phase. In a number of muscles, the peak amplitudes remained well above pre-lesion levels even at the chronic stage. The mean amplitudes of the EMG bursts, EMG burst durations, and total EMG activity per step cycle pre-lesion and post-lesion (normalized to the pre-lesion condition) are shown in Figure 2. In many muscles these three EMG measures showed greater levels of activation post-lesion compared to pre-lesion.

**Figure 3:**
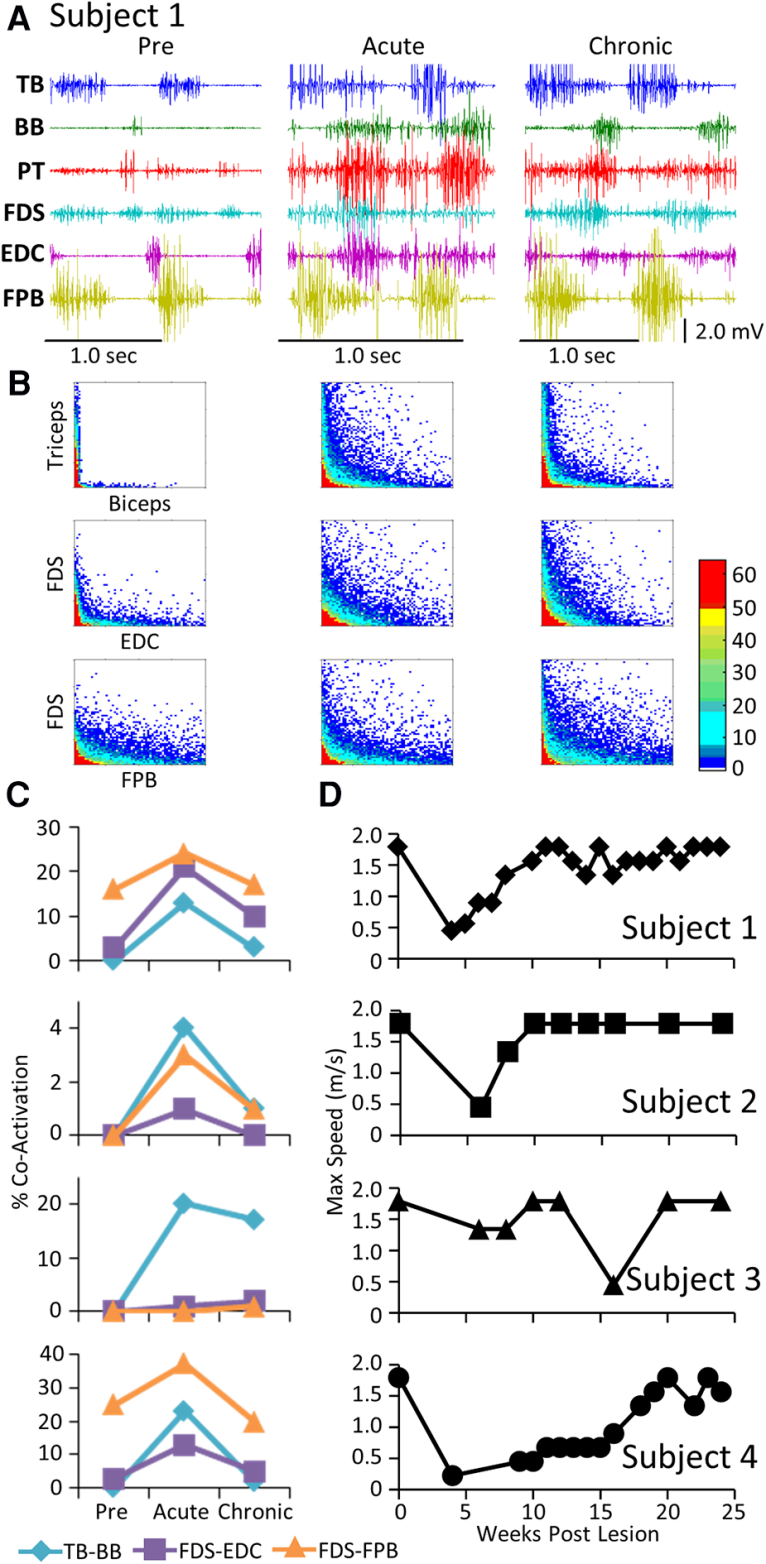
EMG co-activation and locomotor performance. (**A**) Examples of raw EMG activity (2 step cycles for each condition) during treadmill locomotion at 0.89 m/s for Subject 1 for the same muscles and at the pre-lesion and an acute and chronic time points post-lesion. (**B**) Joint probability distributions (see Methods and Materials) of the EMG burst amplitudes between pairs of muscles for the same step cycles shown in (**A**). The y scales are 2 mv for all plots. (**C**) Percent co-contraction for each muscle pair at the pre-lesion stage and at an acute and chronic time point post-lesion. (**D**) Maximum speed of locomotion at each time points for each subject.

The level and pattern of activation in a given muscle represent two of the characteristics that define the output of the muscle. A third factor in defining motor performance is the relative levels of activation of agonist and antagonist muscles, i.e., how well are the activation patterns coordinated. Figure 3 demonstrates a rather consistent pattern in the changes in coordination between pairs of muscles when comparing the pre-lesion and acute and chronic phases of recovery for each subject. The amplitudes of the EMG patterns are consistently elevated post-lesion, particularly in the acute phase as noted above (Figure 3A). The joint probability distributions demonstrate a marked increase in the incidence of co-contraction in agonist and antagonist muscles during locomotion post-lesion (Figure 3B). The percent of co-contraction between each muscle pair studied in Subjects 1, 2, and 4 was higher than pre-lesion at the acute phase and then returned toward near pre-lesion levels at the chronic phase (Figure 3C). The maximum treadmill speed at which each subject could step at each time point was initially decreased but recovered to a near pre-lesion level during the following weeks (Figure 3D).

### EMG activity and force patterns during the GOAS task

**Figure 4:**
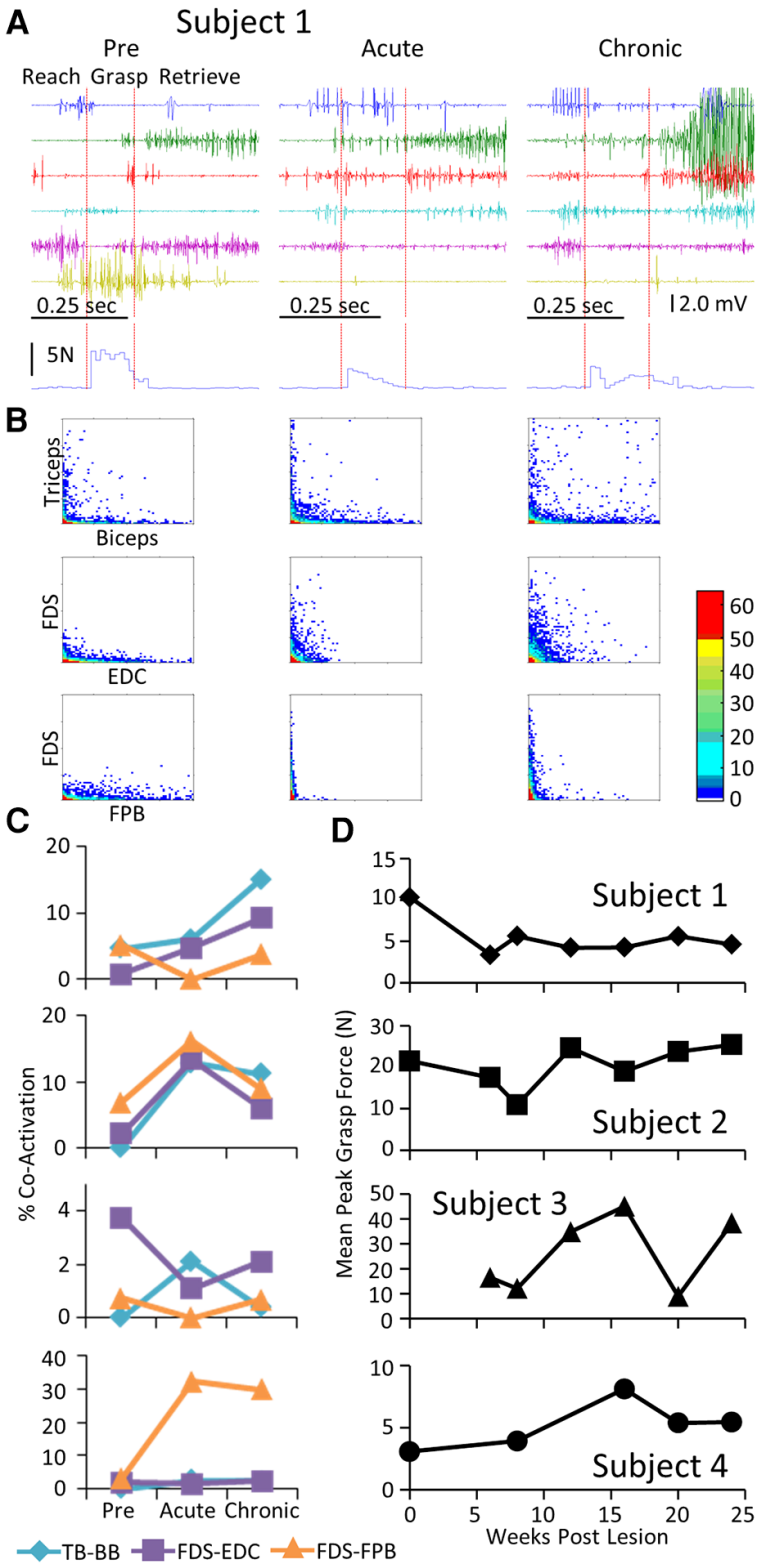
EMG characteristics and performance during the GOAS task. Data for Subject 1 while performing retrieval of a grape placed on a stick instrumented for recording 3-D forces imposed while obtaining the grape presented in a similar format to that in Figure 2. The red vertical lines on the raw EMG graphs in (**A**) divide the task into reach, grasp, and retrieve phases. The resultant of the three force vectors imposed on the stick is shown below the EMG traces. (**B**). Distribution plot of EMG amplitudes of agonist-antagonists muscles (**C**) Percent co-contraction for each muscle pair at the pre-lesion stage and at an acute and chronic time point post-lesion. (**D**) The integral of the force x time for all successful trials for the task for each subject at each time point**.**

Each subject was able to complete the task by the end of the post-lesion period. The regularity with which they could successfully complete the task and the strategy they used to do so, however, varied from subject to subject (Supplementary Video S2). Similar to that observed during the locomotion task, a stereotypical EMG pattern was associated with the pre-lesion performance of the GOAS task. This included (a) an initial activation of the TB and EDC as the subject extended the arm towards the grape (Reach) and opened the hand in preparation for grasping, (b) activation of the PT, FDS, EDC, and FPB to adjust the hand to perform the chosen grasping strategy (Grasp), and (c) prolonged activation of the BB as the subject moved the grape from the stick to its mouth (Retrieve) (Figure 4A). Compared to pre-lesion, the EMG burst amplitude of most muscles was higher at both the acute and chronic stages post-lesion and Subjects 2, 3, and 4 showed higher levels of forces imposed on the stick while retrieving the grape. Concomitant with the increase in EMG amplitudes there was a much higher incidence of co-activation among most pairs of muscles studied post-lesion (Figures 4B,C). In addition, the patterns of changes in co-activation were unique for each subject and the changes in levels of co-activation were generally unrelated to successful performance of the task (compare Figures 4C,D). This variation seems to reflect the unique strategies that each subject adopted to successfully retrieve the grape (see Supplementary Video S2). Although the response varied among subjects, the percent success was near a pre-lesion level by the end of the 24-week recovery period in three of four subjects (Figure 4D).

**Figure 5:**
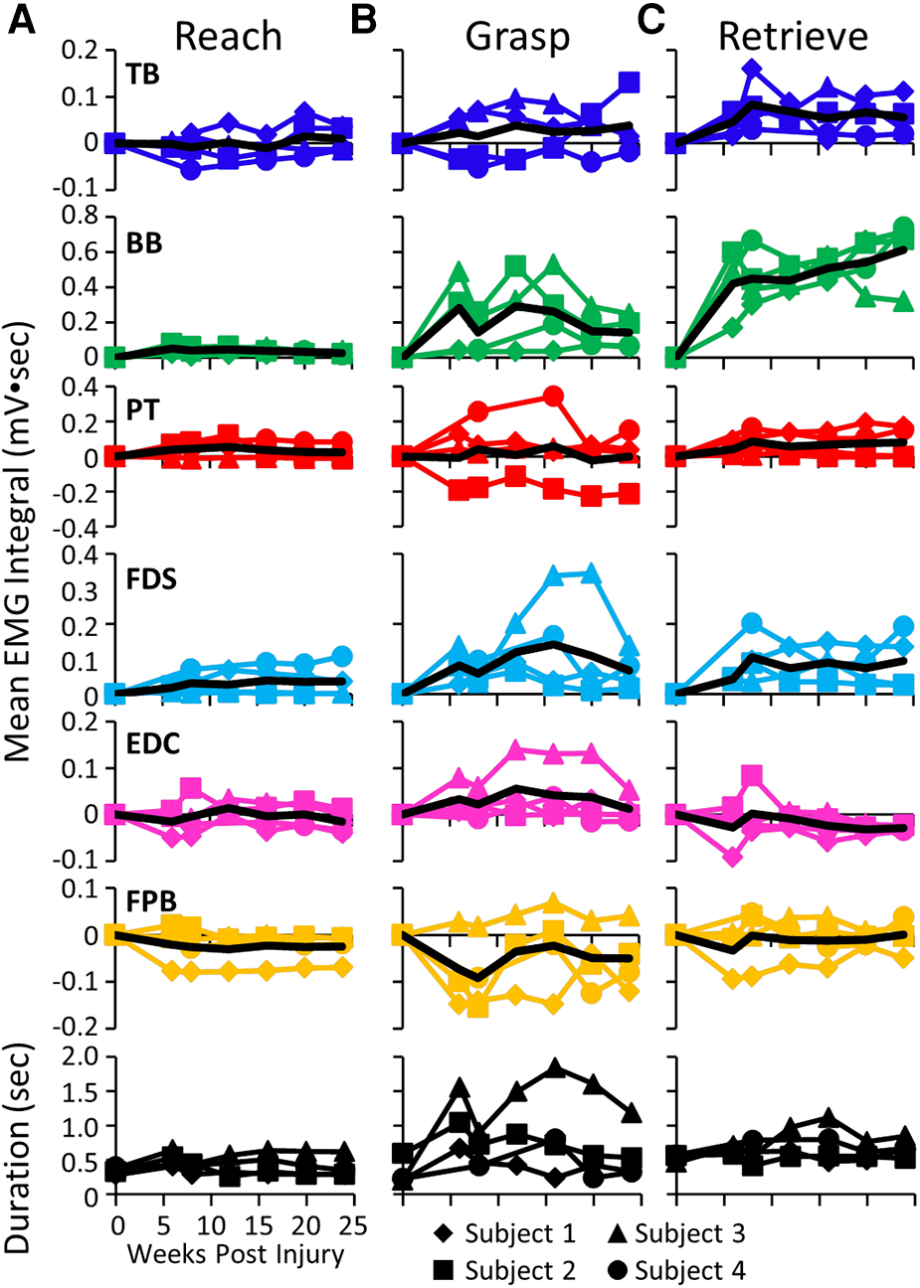
EMG activity during reaching, grasping and retrieving. Mean EMG integrals for each muscle and for each subject and the overall mean EMG integrals (black line) and durations (bottom row) during the reach (**A**), grasp (**B**), and retrieve (**C**) phases of the grape on a stick task. The mean EMG integrals are normalized to pre-lesion values at each time point.

The mean EMG burst integrals during the reach, grasp, and retrieve phases of the GOAS task at all time points are shown in Figure 5. All four subjects showed similar changes in most muscles over the course of the experiment. During the reach phase, the integral values remained relatively unchanged post-lesion (Figure 5A). All integral values throughout the recovery period were elevated in all four subjects in the TB, BB, and FDS during the retrieval phase (Figure 5C). This also was true for the BB and FDS during the grasp phase (Figure 5B). The durations of the reach and retrieval phases remained relatively close to the pre-lesion values throughout the recovery period, whereas the duration of the grasp phase was elevated at most time points post-lesion in all subjects (Figures 5A–C).

### EMG activity and force patterns during the handle pull task

**Figure 6:**
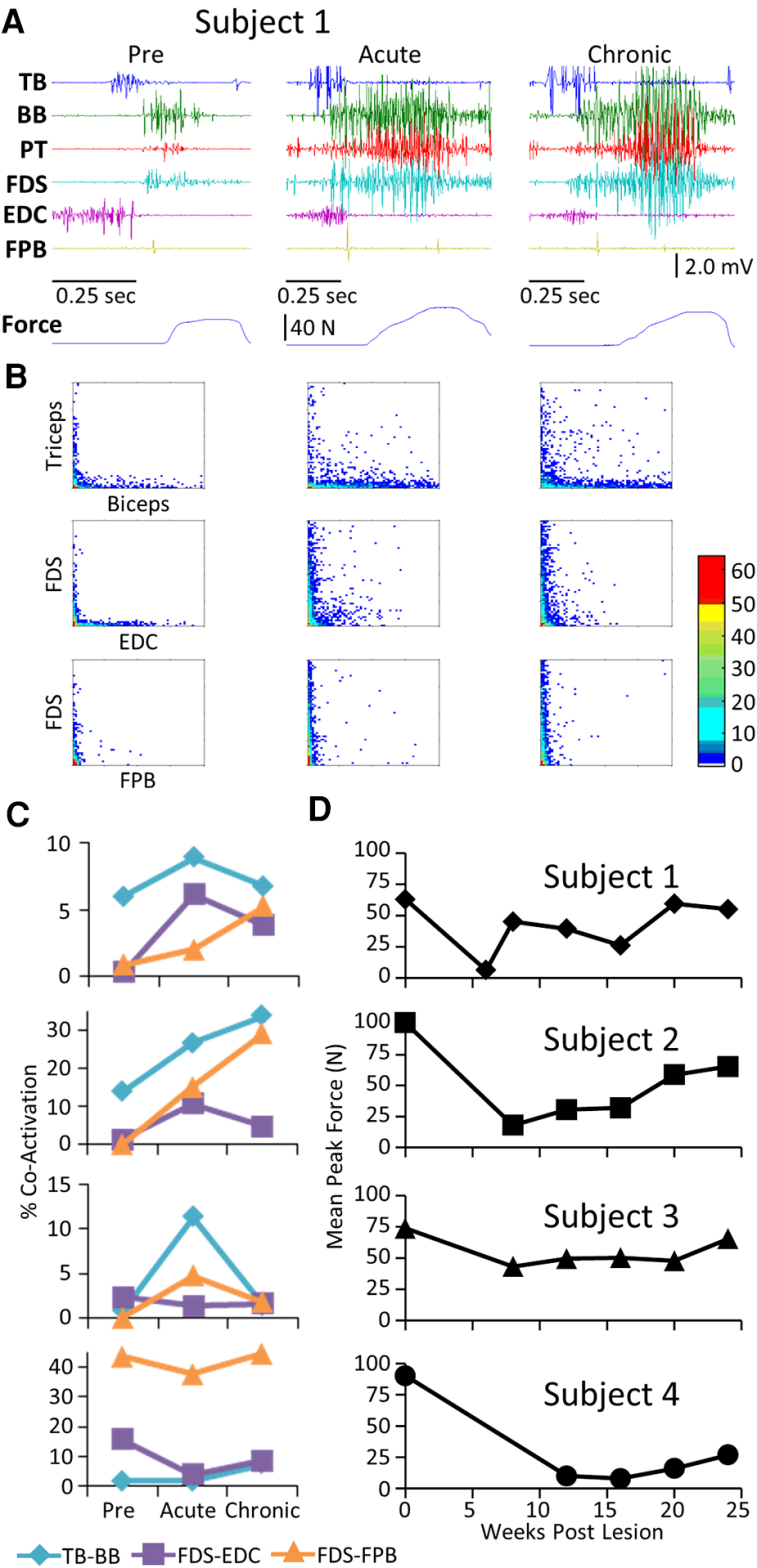
EMG characteristics and performance during the handle pull task. Data for each subject while performing the handle pull task instrumented for recording 3-D forces imposed presented in a similar format to that in Figure 2. Traces of the amount of force generated over the duration of the trial are shown below the raw EMG traces. (**B**) Joint probability distributions of the EMG burst amplitudes between pairs of muscles for the same step cycles shown in (**A**). (**C**) Percent co-contraction for each muscle pair at the pre-lesion stage and at an acute and chronic time point post-lesion. (**D**) The average peak force during the task for each subject at each time point.

Pre-lesion, the stereotypical pattern of EMG activation during the handle pull task included a) activation of the TB and EDC as the subject extended the arm and prepared to grasp the handle, and b) activation of the FDS, BB, and PT as the subject grasped the handle and pulled on it (Figure 6A). As with the other tasks, there was a general increase in the EMG burst duration and amplitude in most muscles of all subjects in the acute and chronic stages post-lesion. There were high incidences of co-contraction (Figure 6B) and this persisted throughout the recovery period in Subjects 1 and 2 but not in Subjects 3 and 4 (Figure 6C). Although the increased incidence of co-activation observed at the acute stage had subsided completely in the TB and BB in Subject 3 at the chronic stage (Figure 6C), the mean maximum force (Figure 6D) in the handle pull task remained high across all time points.

**Figure 7:**
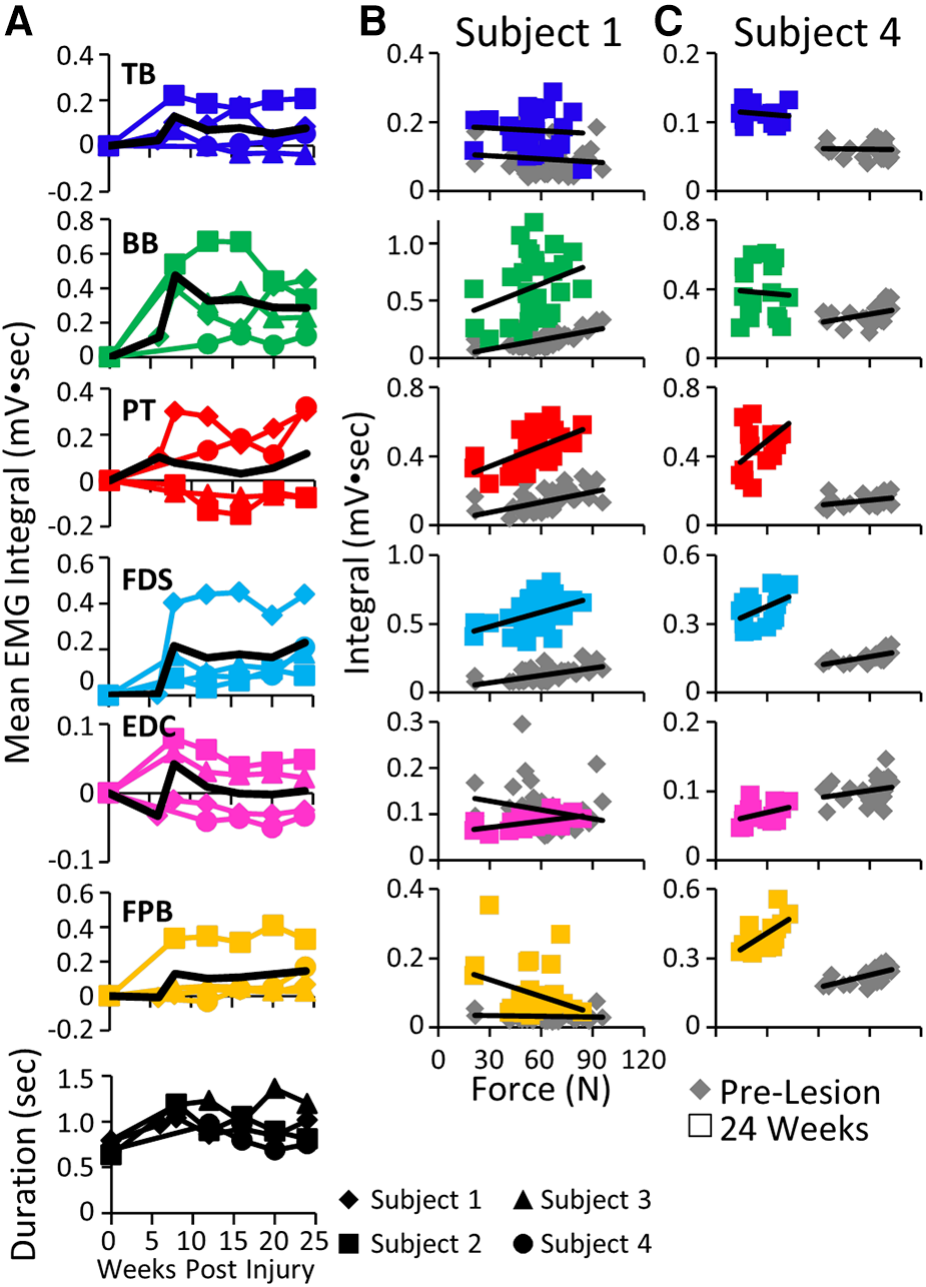
EMG activity and kinetics during the handle pull task. (**A**) Mean EMG integrals for each muscle and for each subject and the overall mean EMG integrals (black line) and durations (bottom row) during the handle pull task pre-lesion and between 6−24 weeks post-lesion. The mean EMG integrals are normalized to pre-lesion values at each time point. (**B**) Relationships between individual force values and the corresponding EMG integrals for each muscle for Subject 1 (**B**) and Subject 4 (**C**) pre-lesion and at 24 weeks post-lesion.

The mean EMG burst integrals were elevated in most muscles in most subjects during the handle pull task post-lesion (Figure 7A). The most common pattern of recovery of EMG patterns and the time taken to complete the task was reaching a peak within about 10 weeks post-lesion and thereafter generally plateauing up to 24 weeks. The forces generated during the task at both the acute and chronic phases of recovery were either elevated or about the same as pre-lesion in Subjects 1, 2, and 3 as illustrated for Subject 1 in Figure 7B. In 5/6 muscles studied in Subject 1 the EMG integral was consistently elevated relative to the force generated (Figure 7B). In contrast, the forces generated in Subject 4 were much smaller post-lesion compared to pre-lesion and there was little relationship between the EMG levels and the forces generated (Figure 7C). Subject 1 was able to successfully complete the spring pull task with the stiffest spring at the pre and all chronic time points, as reflected by the large spread of forces in Figure 7B. Subject 4 was able to pull only the least stiff spring throughout the post-surgical recovery period (Figure 7C).

### Global assessment of the relationship between task performance and the levels of co-contraction of antagonistic muscles over time

To assess the global impact of the post-injury time-point on the relationship between the levels of task recovery and muscle co-contraction we applied non-linear principal component analysis (NL-PCA) separately for the spring, grape-on-a-stick, and locomotion on a treadmill tasks. NLPCA analyzes the cross correlations across all outcomes, combined with optimal scaling transformations, to detect multidimensional patterns using an alternating least squares approach and represent these patterns as synthetic axes known as the principal components (PCs). To understand what the PC axis represents, we calculated the correlation (*PC loading*) of each individual variable with the PC axis. To measure the hypothesized effects of time post-injury on the multidimensional pattern, we calculated the precise *PC score* for each subject.

**Figure 8:**
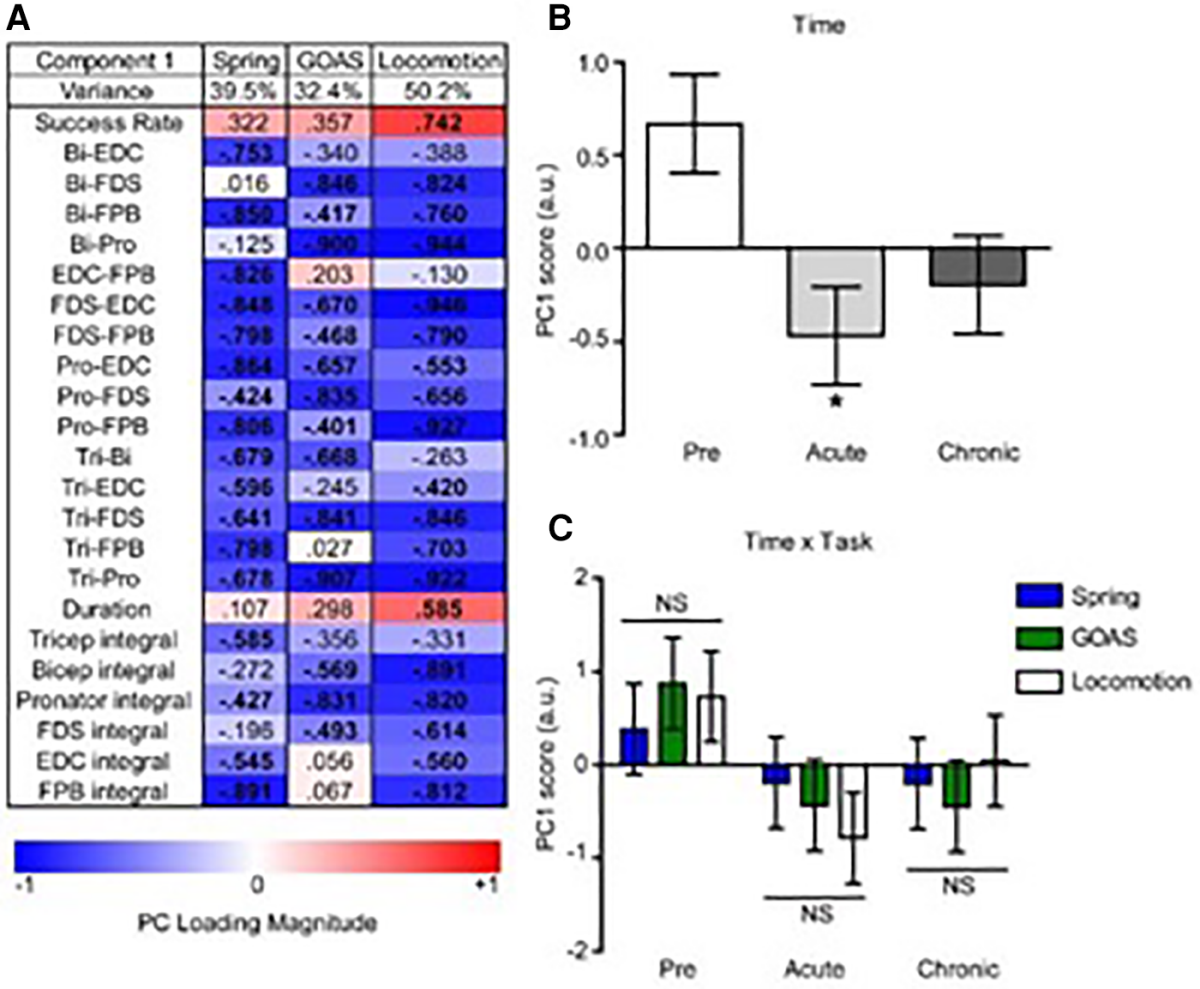
Multivariate analysis of the recovery of behavioral success and muscle co-contraction post-injury. Non-linear principal component analysis (NLPCA) was performed on time series data from muscle integrals and co-contraction distributions during the recovery of success rate for the spring, grape on a stick (GOAS) and treadmill locomotion tasks. (**A**) The first principal component (PC1) accounted for a substantial percentage of the variance in each dataset (39.5%, 32.4%, 50.2%, respectively), and generally represents the inverse relationships between the recovery of task success rate and muscle co-contraction and integrals. PC loadings represent the correlations for each variable onto the entire multivariate PC space (shown separately for spring, GOAS and locomotion tasks): red indicates a positive correlation, blue a negative correlation, and bolded PC loadings (>|0.4|). (**B**) Composite PC scores for each monkey at each time point. A significant main effect of time was found [*F*(2,27) = 4.396, partial *η*^2 ^= 0.246, 1 − *β* = 0.709, *p* = 0.022], with the acute time point showing a significant decrease compared to pre-injury (*p* < 0.05), and recovering towards the chronic phase that was not significantly different from pre-injury. (**C**) Two-way GLM ANOVA showed no significant time by task interaction [F(4,27) = 0.443, partial *η*^2 ^= 0.062, 1 − *β* = 0.137, *p* = 0.777]. Histograms are plotted as mean ± standard error. *p < 0.05. NS, not significant.

PC1 loadings revealed an inverse relationship between recovery success and muscle co-contraction for each task (Figure 8A). Analysis of PC scores by time point-post injury revealed a significant global pattern of co-contraction (Figure 8B) associated with impairments in function at the acute post-injury time point that were partially restored at the chronic time point. There was not a significant difference between the tasks in this global pattern, i.e., the time x task interaction was not significant (Figure 8C).

## Discussion

### Motor performance after a partial spinal cord injury (hemisection) was limited by poor coordination of motor pools

The present results suggest that the limitations in performance of the motor behaviors studied, once beyond the acute stage of the injury, were more closely linked to the abnormal levels of coordination among the motor pools rather than the inability to recruit the motor pools. In most cases the motor pools were much more active post-lesion compared to pre-lesion in each of the motor tasks studied. Although some muscles continued to be activated at relatively low levels during some of the tasks, this particular factor alone did not seem to be a major limitation in task performance. An important implication of these changes in coordination among the motor pools combined with the elevated levels of activity was that there appears to be a significant neural reorganization of functional connectivity at one or more points along the sensorimotor axis extending from the sensorimotor cortex to the spinal motor pools as a result of a partial spinal cord injury (22, 23).

This elevated activation level of motor pools in response to supraspinal and sensory input are consistent with the observation that motor-evoked potentials induced with transcranial simulation in human subjects after a partial cervical spinal cord injury continued to increase in amplitude when generating up to 50% maximum voluntary contraction whereas the amplitude plateaued at about 10% of maximum voluntary contraction for control subjects (7, 24). The increased muscle activation after the partial spinal cord injury observed in the present study also is consistent with the observation that although spontaneous cage activity was relatively low after a spinal cord injury in rats, increasing the excitability level of the lumbosacral spinal cord networks to ∼20% below motor threshold via epidural stimulation increased cage activity five-fold (25). These observations, combined with the present data, are consistent with the resting level of excitability of the sensorimotor networks being relatively low after paralysis (14, 26), but once the motor threshold is reached the aberrant connectivity can lead to greater excitatory input to the motor pools.

#### Recovery remains continuously dynamic for months after a spinal cord injury

In the acute stages after a spinal cord injury there is a period of days to weeks in which the excitatory state of the spinal circuitry below the lesion is relatively quiescent electrically, a fundamental property of “spinal shock”. In the present study, over a period of two months there was a gradual and substantial recovery in the ability to activate groups of muscles resulting in the generation of considerable forces. The activation levels of the muscles were routinely higher post-lesion than pre-lesion. What are the neural mechanisms that could underlie this elevated activation of motor pools post-injury? From a fundamental physiological perspective this must involve activation of more motor units and/or activation of motor units at a higher frequency within each burst of activity. We suggest that the elevated EMG can most logically be explained by the greater excitability derived from more motor units being activated and not more action potentials generated per motor unit.

An unusually high level of functional connectivity among interneurons that directly or indirectly project to motoneurons could explain, at least in part, the observed increase in motor unit activity (8). The higher level of co-activation between agonists and antagonists also would be a predictable outcome from newly formed functional connections, particularly if there was a proliferation of aberrant supraspinal and/or spinal networks that directly or indirectly project to cervical spinal motor pools that are not normally functionally connected. As functional reorganization of connectivity also could explain the present observations reflecting changes in the reorganization of the neural control for stepping, as well as for tasks requiring fine motor control of the hand. There were no consistent differences in the changes of aberrant connectivity observed either during locomotion, retrieving a grape, or pulling on a handle, three tasks differing in the degree of automaticity required to perform the tasks, i.e., fine motor control of the more distal muscles vs. stepping on a moving treadmill belt. These data are consistent with there being a proliferation aberrant connectivity formed within or among supraspinal and/or spinal networks.

Other observations are consistent that a loss of precision in the temporal modulation and levels of activation of different motor pools contributed to the formation of functionally aberrant connectivity within the spinal networks. For example, after a complete mid-thoracic spinal cord transection in adult rats and cats, there is a significant increase in the amount of co-activation between flexors and extensors during stepping (16, 27–30). After weeks of step training of these animals, however, the incidence of co-contraction among agonists and antagonists can be reduced significantly. Thus, the spinal networks controlling stepping can function with near-normal coordination patterns without any supraspinal control after a complete spinal cord transection, whereas there is clearly a greater dependence on supraspinal control when performing the GOAS and handle pull tasks after a spinal cord hemisection. The similarity in the degree of functionally elevated aberrant connections during each motor task performed in the present study leaves the possibility that the abnormality of the connectivity observed could be attributed largely to spinal networks, particularly during locomotor tasks. Given the multiple examples of cortical and brainstem reorganization reflecting the formation of aberrant connectivity and elevated neuronal activity after an injury, however, it seems likely that the supraspinal networks also played some role in shaping the motor recovery observed in the present study (31–33).

#### What Are the structural mechanisms associated with the continuously adapting functional connectivity

What could be the mechanisms for the changing functional connectivity among neurons? Commonly used terms and concepts that are presumed to be the basis of neural reorganization include “collateral sprouting” of uninjured axons and dendrites and “axonal re-growth” of injured neurons, each of which could occur concomitant with a proliferation of synaptic inputs along the sensorimotor axis at one or more sites (34). Other possibilities could be less anatomically based, such as rapid changes in the efficacy of synaptic connections. Although there are multiple mechanisms that might underlie the observed changes post-injury, we cannot attribute the changes in functional connectivity to a specific anatomical mechanism. Based on the present data we propose that a common mechanism for elevated neuromuscular activity of the spinal circuitry when performing a motor task after a spinal cord injury is attributable to the relatively widespread development of functionally novel, aberrant connections as well as some that become more functionally useful via activity-dependent mechanisms. For example, in subjects with a central cord syndrome, a cervical spinal cord injury that affects the upper limbs more than the lower limbs, clear examples of the loss of specificity in being able to voluntarily activate only the intended muscles in response to transcranial magnetic stimulation was observed as early as three days post-injury (4). More specifically when the subjects were asked to activate the left adductor pollicis brevis, there was simultaneous EMG activity in the left extensor carpi radialis and right abductor hallucis muscles followed by the left abductor hallucis, right quadriceps, and soleus muscles. These types of nonspecific activation are rarely observed in able-bodied subjects. As stated by these authors, these results demonstrate a “diffuse excitation of nonspecific populations of interneurons and motoneurons”.

The present data demonstrate that although initially there was an extensive development of newly formed connectivity there also was a continuous reshaping of a less diffuse connectivity over time. In some cases nearly normal functionally coordinated patterns returned by 24 weeks post-lesion, although to some degree the functionally aberrant connectivity persisted among most motor pools. We propose that aberrant functional connections contributed to the loss of specificity in the ability to activate the appropriate combination of motor pools and thus the poor coordination of motor pools when attempting to perform a specific task. We also have described this loss of specificity in chronic spinal cord injured patients. For example, when instructed to move a specific joint there was a simultaneous activation of flexors and extensors across multiple joints within a limb and even in both limbs (9).

Although it is clear that some of this adaptation in circuitry could be facilitated directly to individual motor pools, it is highly likely that mediation via interneurons will be effective in establishing more normal patterns among motor pools. This interpretation is consistent with the multiple experiments extensively reviewed by (35) and to experiments involving, full load-bearing stepping over a range of speeds, directions of stepping based on body orientation to the treadmill belt, including the reversal of the treadmill belt in animals that have a complete mid-thoracic spinal cord transection (15, 36, 37). In these latter experimental preparations, the only source of control had to be derived from the total ensembles of proprioception derived from a multitude of mechanoreceptors from the neuromuscular system of the hindquarters of the animal.

Results from studies by Bizzi and colleagues (38) and Lacquaniti and Ivenenko (39), as well as from our lab (28) have demonstrated the dominate role that the spinal circuitry can play in controlling locomotion without any influence from supraspinal control. Thus, the key question remains: “what is the anatomical-biochemical substrate of these functionally aberrant, as well as useful, connections that seem to emerge so quickly?” Given our observations of the recovery of voluntary movements within a few weeks after the initiation of epidural stimulation below the lesion in four human subjects with chronic motor complete paralysis, some neural connectivity might emerge initially from functionally incompetent neurons (34). While actual growth of axons in a more macro-perspective to new targets is theoretically possible, the speed at which these changes occurred in these paralyzed subjects and the spread of the activation patterns from a unilateral to a bilateral one as reported by Alexeeva et al. (4) makes this possibility seem less likely. Similar phenomena of forming aberrant projections at the supraspinal level have been reported (31).

We do know from studies of recovery of locomotion after a complete spinal cord injury that as the animal improves in its ability to step there is a concomitant improvement in the pattern of reciprocity of activation of agonist and antagonist muscles (16, 28, 40). A decrease in the incidence of co-activation over time occurred in the present study in most muscles over a 24-week recovery period. Given that the pattern and timing of activation of multiple motor pools involved in the movement are determined largely by the interneurons, this gradual reduction in co-contraction seems likely to be a consequence of some type of functional “pruning” of synaptic connectivity among interneurons and their eventual projections to motoneurons (41, 42).

The present data provides a comprehensive ensemble of observations which formulates a systems level biological concept which reflects a highly integrated plastic response to a severe neuromuscular injury. The nature of this response has significant implications for the rehabilitative strategies that we embrace following such injuries. First, the potential for recovery of function given the apparent level of plasticity, particularly in the neuromuscular systems are clearly evident. Multiple examples supporting this general concept are evident in Figure 1. For example in almost all cases, the amplitude of the EMG during locomotion is significantly higher beginning around six weeks post-lesion and this elevation persist in almost all muscles in each of the subjects. In addition, during this 6 to 24 week some muscles shows a gradual decline, but remains higher than the pre-lesion state. For example, the amplitude of the FDS and EDC in subject one declines between after 20 and 24 weeks. In addition, the temporal pattern of the EMG changed markedly in the EDC as well as the time of the peak EMG amplitude, demonstrating a change in how a specific muscle can be coordinated which probability of change in the mechanics of the movement unless there is another muscle that changes in a manner that compensates for the changes in another motor pool. Further important implications of these variations in patterns of EMG during locomotion is that many combinations of activation patterns of the motor pools can be recruited with little change in the mechanics of the movement, i.e., there are many ways for the nervous system to generate different patterns of activation of multiple motor pools in the uninjured as well as in the injured state. This is a common observation when changes the mechanics of stepping in response to different acute and chronic perturbations, such as simply injuring the foot or as occurs during normal aging. The importance of this concept in rehabilitation is that when some of these options are eliminated due to some pathology or trauma, there remains alternate patterns that can achieve virtually the same or at least a similar outcome and thus should be taken advantage when possible.

The concept noted above is also relevant from a biochemical perspective in that the performance of a given motor task reflects a functional change in network by changing the activities of selected chemoreceptors, such as serotonergic subtypes that respond to a specific presynaptic transmitter. The classic example, is the monosynaptic circuit in which there is not only monosynaptic activation of motor neurons but simultaneously there are interneurons that inhibit antagonistic motor neurons. It has been demonstrated in many cases for example improve stepping via disinhibition. A dramatic demonstration of this phenomenon was to transform the physiological states of the spinal circuitry in an animal that cannot step following a complete transaction regained full weight bearing stepping within 30 min following administration of a pharmacological disinhibitor such as strychnine (16, 43). The mechanism for the spinal networks to achieve this inhibitory effect normally is via glycinergic or gabaergic neuro transmission. This type of circuitry is a key component of interneurons of central pattern generation. But they play important roles in defining the temporal patterns of activation of most motor pools. This temporal pattern of activation of motor pools defines the pattern that generates every motor behavior (34, 43, 44, 45). In rehabilitation one of the key objectives in many cases is to facilitate adaptations toward a more functional balance of inhibition and excitation of agonists and antagonist motor pools and thus the muscle which they innervate.

#### Clinical implications

The present observations following a partial spinal cord injury demonstrate (1) initially, a marked loss of the ability to activate the appropriate muscles, (2) the subsequent formation of excessive and aberrant inter-neuronal connections within the spinal and/or supraspinal circuitry, and (3) finally a gradual reduction, i.e., “pruning” of these aberrant connections. Understanding the systems-level mechanisms that could generate this kind and level of spontaneous motor recovery after a cervical spinal cord lesion could be critical in the development of interventions that can facilitate recovery of function. More specifically, the question becomes “to what extent can the early post-injury quiescent phase be followed by the subsequent formation of more functional but less aberrant connections, thus, maximizing recovery by enhancing recruitment levels of motor pools, while also sustaining more normal coordination among motor pools”?

Previous studies of the recovery of stepping after a complete mid-thoracic spinal cord transection suggest that improvements in the coordination of motor pools can be attributable to training (37, 41, 46–48). Accordingly, we propose that activity-dependent-learning mechanisms, as occurs during training, will facilitate pruning and functionally selective guidance of supraspinal and spinal network connectivity leading to more effective coordination among motor pools (49). We further suggest that a major source of guidance in generating more functionally significant connectivity that leads to better coordination is derived from ensembles of proprioceptive input to the spinal interneurons that play a major role in defining the patterns of activation among multiple motor pools.

## Data Availability

The original contributions presented in the study are included in the article/**Supplementary Material**, further inquiries can be directed to the corresponding author.
