# Supplementary figures and images for: Emergence of functionally aberrant and subsequent reduction of neuromuscular connectivity and improved motor performance after cervical spinal cord injury in Rhesus
Source: Front Rehabil Sci. 2023 Jun 12;4:1205456. doi: 10.3389/fresc.2023.1205456 (PMC10291623; doi:10.3389/fresc.2023.1205456)

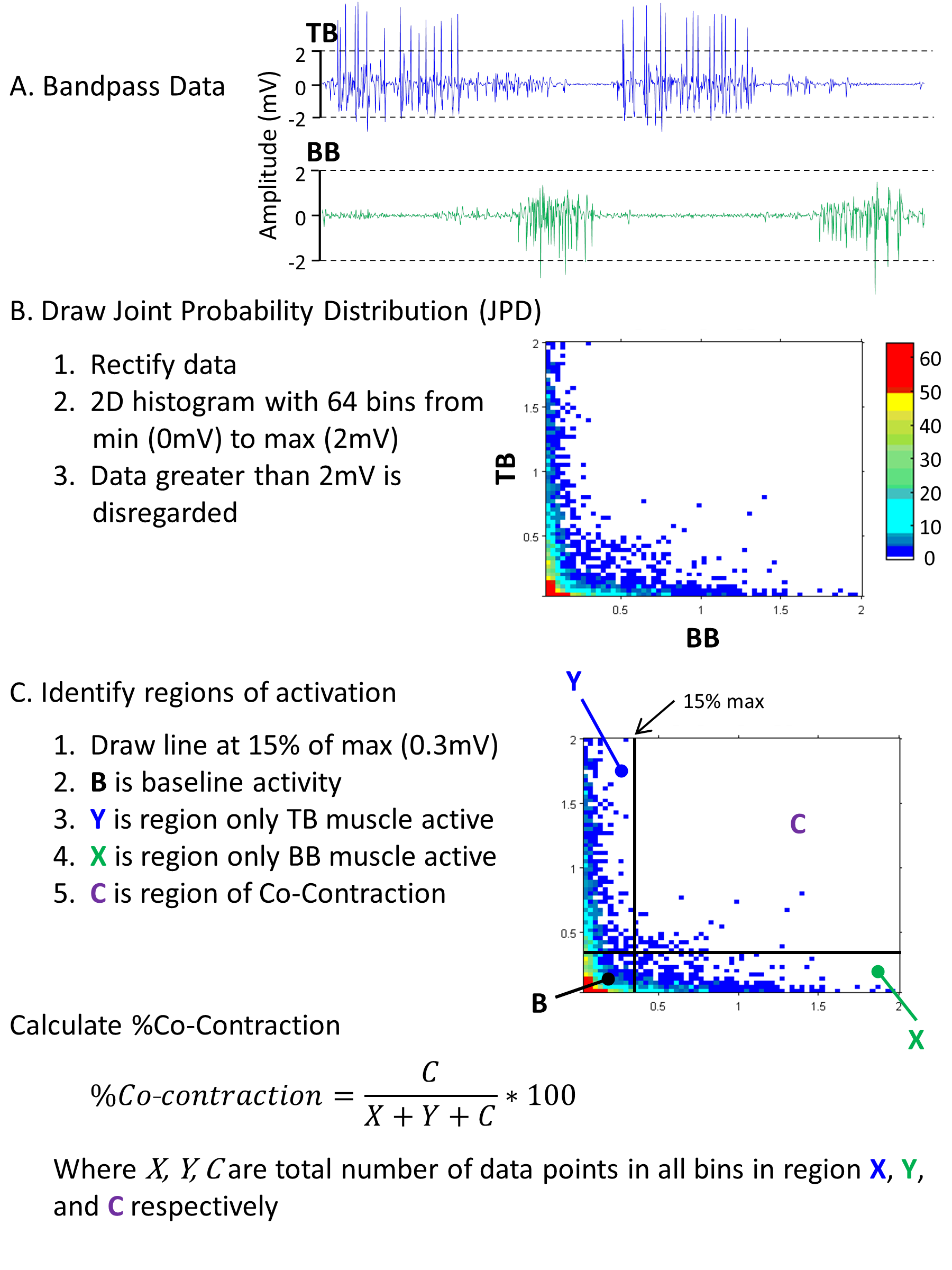

Supplement: Supplementary file 1 [file Image1.tif]
